# Enigmatic declines in bird numbers in lowland forest of eastern Ecuador may be a consequence of climate change

**DOI:** 10.7717/peerj.1177

**Published:** 2015-08-11

**Authors:** John G. Blake, Bette A. Loiselle

**Affiliations:** 1 Department of Wildlife Ecology and Conservation, University of Florida, Gainesville, Florida, USA; 2 Department of Wildlife Ecology and Conservation and Center for Latin American Studies, University of Florida, Gainesville, Florida, USA

**Keywords:** Birds, Conservation, Long-term, Population declines, Tropical, Birds, Ecuador, Neotropics, Climate change

## Abstract

Bird populations have declined in many parts of the world but most of those declines can be attributed to effects of human activities (e.g., habitat fragmentation); declines in areas unaffected by human activities are not common. We have been sampling bird populations at an undisturbed site in lowland forest of eastern Ecuador annually since 2001 using a combination of mist nets and direct observations on two 100-ha plots. Bird numbers fluctuated on both plots during the first 8 years but did not show a consistent pattern of change. Since about 2008, numbers of birds on both plots have declined; capture rates in 2014 were ∼40% less than at the start of the study and observation rates were ∼50% less. Both understory and canopy species declined in abundance. Overall, insectivores showed the most pronounced declines but declines varied among trophic groups. The period from 2008 onward also was a period of stronger La Niña events which, at this study site, are associated with increased rainfall. The mechanism for the declines is not known but likely reflects a combination of reduced reproductive success coupled with reduced survival associated with changing climate.

## Introduction

Bird populations can vary in abundance over time and space because of variation in rainfall, changes in resources, alteration of habitat, and other factors (Blake et al., 1994; Ballard et al., 2003; Jones, Doran & Holmes, 2003; Stouffer et al., 2011; Shaw et al., 2013). Much attention has focused on long-term declines in migrant populations both on breeding and wintering grounds (e.g., Ballard et al., 2003; Faaborg et al., 2013). Much less attention has been given to long-term changes in populations of tropical species (Latta et al., 2011); few studies have followed populations on an annual basis using consistent methods, although the need for such studies has been recognized (Harris et al., 2011).

The idea that tropical forests are relatively stable environments has a long history (Dobzhansky, 1950); the view of tropical regions as stable and benign climatically led to the idea that populations would be more stable as well (MacArthur, 1955). Although there is some evidence for stability in, for example, territories and flock structure (Martinez & Gomez, 2013), other studies have demonstrated considerable variation in population sizes, community composition, and life-history traits over different spatial and temporal scales (Loiselle & Blake, 1992; Brawn et al., 2011; Latta et al., 2011). Further, rainfall in most tropical regions varies seasonally and over longer time scales (Condit et al., 2004; Dejean et al., 2011) so tropical environments are not necessarily stable. Given that even small changes in humidity may affect distribution patterns on a local scale (Karr & Freemark, 1983), it would not be surprising for distribution and abundance patterns to be affected by longer-term or larger spatial scale variation in rainfall or other climatic factors.

Most studies on declining populations of tropical bird species have focused on effects of fragmentation, and other types of habitat alteration (Robinson, 1999; Robinson, 2001; Stouffer et al., 2011; Shaw et al., 2013). Yet, large-scale climatic cycles, such as El Niño-Southern Oscillation (ENSO) can affect bird populations over large regions, with effects varying geographically (Ballard et al., 2003; LaManna et al., 2012). Changes in rainfall patterns may affect resource abundance (Sillett, Holmes & Sherry, 2000; Jones, Doran & Holmes, 2003) with noticeable effects on survival, fecundity, and other life-history traits (Sillett, Holmes & Sherry, 2000; Nott et al., 2002; Mazzerolle et al., 2005; LaManna et al., 2012), although not all species are affected (Brawn et al., 2011; Styrsky & Brawn, 2011).

Studies on effects of climate change on tropical birds often have focused on changes in distributions along elevational gradients (Forero-Medina et al., 2011; Harris et al., 2011; Anderson et al., 2013; Jankowski et al., 2013; Freeman & Freeman, 2014). Less attention has been given to the possibility that climate cycles might affect bird populations in lowland forests (e.g., Styrsky & Brawn, 2011; Wolfe, Ralph & Elizondo, 2015). Similarly, although many studies have examined changes in bird populations in areas directly affected by human activities (e.g., fragmentation and loss of forest; see above), few long-term studies have been conducted in tropical lowland forests that have not been affected to a noticeable extent by human activities (Martinez & Gomez, 2013). Yet, even “undisturbed” forests may be affected by large-scale climatic cycles such as ENSO and, thus, may still be affected indirectly by human activities if those activities affect climate cycles. Detecting any such effects requires long-term studies that examine bird populations on an annual basis with consistent sampling protocols. Without such studies, much variation in populations may either not be recorded or, if studies are too short, may provide incomplete pictures of the extent of variation given that populations may change in response to short-term changes in environmental conditions or to longer (decadal) patterns in climate.

Long-term changes in species abundance and occurrence may occur even in the absence of obvious anthropogenic disturbances. For example, there was a 75% decline in abundance of leaf-litter amphibians and reptiles between 1970 and 2005 at La Selva Biological Station, Costa Rica (Whitfield et al., 2007). The most likely explanation for the decline was a reduction in standing leaf litter as a consequence of long-term climate change. Such declines in species found in habitats that have not experienced significant, apparent human-driven modifications have been referred to as “enigmatic” declines (Stuart et al., 2004), as they occur in habitats not normally thought of as susceptible to change.

Here, we use data gathered over a 14-year period to examine changes in bird populations in an apparently undisturbed forest of lowland eastern Ecuador. In particular, we focus on substantial declines in overall bird numbers that have occurred over the last 5 to 6 years and discuss possible causes for those declines.

## Methods

### Study site

Research was conducted at Tiputini Biodiversity Station (TBS), Orellana Province, Ecuador (*ca* 0°37′S, 76°10′W, 190–270 m above sea level). TBS is located adjacent to Yasuní National Park on a tract of largely undisturbed (by human activities) lowland rain forest within Yasuní Biosphere Reserve, one of the most diverse regions of the world (Bass et al., 2010). The biosphere reserve encompasses Yasuní National Park (∼982,000 ha), the adjacent Waorani Ethnic Reserve (∼800,000 ha), and a 10 km buffer zone. TBS is located approximately 22 km east of an offshoot of the Maxus Road, ∼25 km east of the nearest Waorani settlement, ∼12 km from an oil access road to the northwest, ∼20 km south of the Napo River, and approximately 17–19 km from the nearest settlements along the Napo River. Thus, the station is surrounded by extensive areas of intact forest. The station and nearby areas are dominated by *terra firme* forest; *várzea* forest, palm swamps, and various successional habitats also are present. Mean annual precipitation at Yasuní Research Station, approximately 28 km west of TBS, is about 3,100 mm.

Two *ca* 100-ha plots (*ca* 1 km × 1 km each) were established in *terra firme* forest during 2001. Both plots are gridded (100 × 200-m grid lines) and marked with 1.5-m PVC tubes. The Harpia plot ranges from ∼201 to 233 m elevation and is characterized by more dissected upland forest. The Puma plot is flatter overall although elevation range is similar, from ∼209 to 235 m. Both areas experience partial inundation when small streams back up as the Tiputini River rises; Puma has more areas that fill with persistent standing water during the rainy season. Dominant vegetation on both plots is tall, evergreen forest.

### Bird sampling

#### Mist nets

Birds were captured with mist nets (12 × 2.6 m, 36-mm mesh) set at ground level. Nets were arranged in a series of eight sets of 12 nets on each plot (96 sites per plot). Each set of 12 nets formed a rectangle (100 × 200 m) with nets set ∼50 m apart; maximum distance between nets on a plot was approximately 920 m. Each set of nets was run for one day (∼0600 to 1200 h) in January (peak of breeding for many species) and one day in March (late breeding season for many species), starting in March 2001 and ending in March 2014. Captured birds were identified and most (except some hummingbirds) were banded with aluminum leg bands. See Blake & Loiselle (2009) for more details on netting protocols.

#### Direct observations

One of us (JGB) sampled birds during February 2003–2006, 2009, 2010, 2013, 2014. Locations of all birds seen or heard while walking along transects were noted on scale maps of the plots; unknown songs were tape-recorded for later identification. Approximately 1–1.4 km of transects were covered during a morning; starting positions were distributed throughout the plots to ensure, as much as possible, that all parts of the plots were covered early in the morning when vocal activity is greatest. Each plot took ∼12–13 days to cover; transects were not walked more than once during a given sample. Total effort expended (i.e., numbers of hours and numbers of kilometers) was equivalent between plots and among samples, except in some later samples when heavy rains interrupted samples. Counts started well before light, when the first diurnal birds were beginning to sing and when many nocturnal species were still vocalizing. Vocal activity typically was high until ∼2 h after sunrise, when it often declined rapidly; thus, counts were confined to the first few hours of the morning. Periods of rain occasionally interrupted or ended counts early.

Not all species or groups of species were equally well sampled. For example, some canopy tanagers (*Tangara* spp.), small flycatchers and hummingbirds in the canopy likely were often overlooked, as were other canopy birds that do not vocalize much (e.g., some puffbirds). In contrast, highly vocal species with distinctive songs likely were not missed and may be relatively over-represented. Yet, because counts were conducted in the same way on both plots, between-plot comparisons and among-year comparisons should not be affected. Hawks, psittacids, and other species flying above the canopy were noted but not included in most analyses if they were not observed using the plot. Swifts and swallows were seen flying over the forest but numbers were not estimated and are not included in results. See Blake (2007) for more details on observation methods. Taxonomy follows Remsen et al. (2015).

### Analyses

Sample effort (number of mist-net hours, where one mist net open 1 h equals 1 mist-net-hour or 1 mnh) varied slightly among samples. Consequently, comparisons of numbers of captures were based on captures per 100 mnh. Similarly, observations were expressed as number of birds per 100 m or 1,000 m to standardize numbers by effort. Species were classified into foraging guilds using categories of Terborgh et al. (1990). We used correlation coefficients to examine patterns of change in overall abundance, abundances of different guilds, and abundances of individual species represented by at least 140 captures or observations (or at least 100 on a single plot). We used correlation coefficients to examine patterns of change in relation to year and to Southern Oscillation Index (SOI) values with and without a 1 or 2-year lag. We used annual SOI values and values from October to March, a period that encompasses the main breeding period at TBS and that overlaps the sampling period (January through March). We used 1- and 2-year time lags under the assumption that if changes in climate affected reproduction and/or survival, those effects might not be felt immediately but rather would accumulate over time. SOI values were obtained from National Weather Service, Climate Prediction Center, National Oceanic and Atmospheric Administration (http://www.cpc.ncep.noaa.gov/data/indices/). Comparisons between plots were based on correlations and paired *t*-tests as appropriate. Correlations are used to indicate strength of association, without necessarily attributing a *P*-value. All statistical analyses were run with Statistix 10.0 (Analytical Software, 2013).

Approval for this research was obtained from Non-Regulatory Animal Research Committee (018-10WEC) at University Florida and, previously, from IACUC, University of Missouri. Work at Tiputini Biodiversity Station was conducted in accordance with research permit number 13-IC-FAU-DFN (and subsequent renewals), Ministerio del Ambiente, Distrito Forestal Napo, Tena, Ecuador.

## Results

### Overall summary

We accumulated 7,302 captures (2,780 recaptures) of 155 species in 14,862 mnh on Harpia and 7,598 captures (2,938 recaptures) of 154 species in 14,263 mnh on Puma; 178 species were captured in total. We did not use mist nets to sample birds on either plot during March 2013 or on Puma during 2014 because heavy rains reduced our opportunities for netting. We accumulated 20,391 records of 276 species on Harpia and 17,427 records of 275 species on Puma; 306 species were recorded on both plots combined.

### All species combined

**Figure 1:**
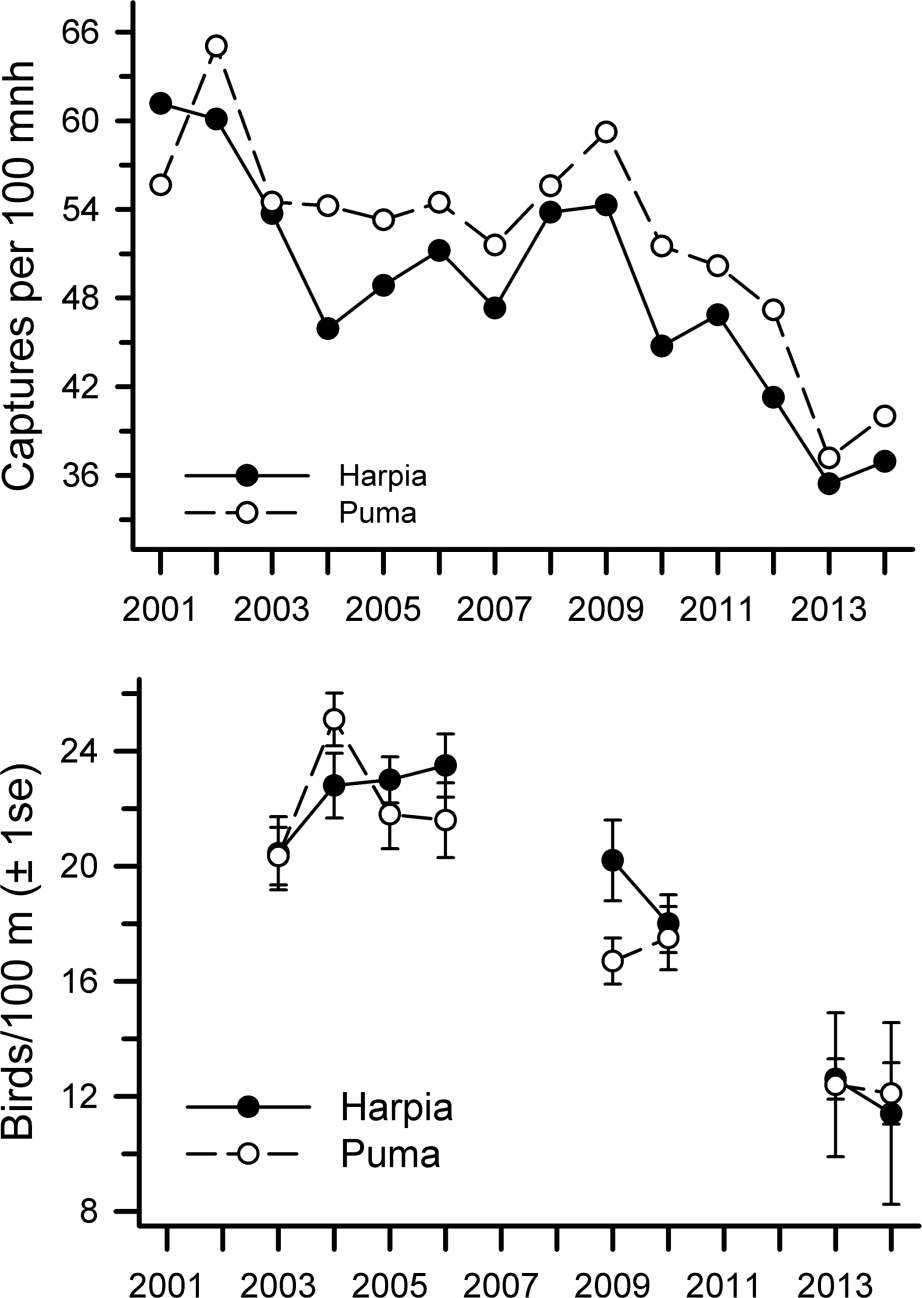
Capture rates and observations of birds at Tiputini Biodiversity Station. Capture rates (A) and observations (B) of birds on two 100-ha plots (Harpia, Puma) at Tiputini Biodiversity Station, Ecuador, from 2001 to 2014.

Annual capture rates (birds 100-mnh^−1^) were highly correlated between plots (Table 1) but were somewhat higher overall on Puma (Harpia: mean = 48.7 ± 2.1 SE, CV = 15.8; Puma: 52.1 ± 1.9, CV = 13.7; paired *t*-test, *t* = 3.91, df = 13, *P* < 0.002). Although overall capture rates declined from 2001 to 2014 on both plots (Table 1), declines were most pronounced from 2009 to 2014 (*r* = − 0.47 and −0.23 from 2001 to 2009 on Harpia and Puma respectively; *r* = − 0.92 and −0.94 from 2009 to 2014 on Harpia and Puma, respectively) (Fig. 1A). Capture rates on Puma in 2009, for example, were as high as they were in 2001.

**Table 1:** Correlations between bird numbers and years and between plots. Correlations in capture and observation rates of guilds across years and between plots. Guilds follow Terborgh et al. (1990). *P*-values are given without correction for multiple comparisons.

	Across years				Betweeen plots	
Group	Harpia		Puma		
	*r*	*P* <	*r*	*P* <	*r*	*P* <
**Captures**						
All species combined	−0.83	0.001	−0.77	0.002	0.90	0.001
Mixed-species flocks	−0.59	0.03	−0.50	0.07	0.57	0.04
Arboreal insectivores	−0.76	0.002	−0.60	0.03	0.61	0.03
Gleaning	−0.69	0.006	−0.60	0.03	0.60	0.03
Sallying	−0.71	0.005	−0.39	0.17	0.47	0.09
Dead leaves	−0.43	0.13	−0.77	0.002	0.68	0.01
Terrestrial insectivores	−0.76	0.002	−0.82	0.001	0.92	0.001
Army-ant followers	−0.53	0.05	−0.50	0.07	0.35	0.21
Arboreal frugivores	−0.59	0.003	−0.60	0.03	0.55	0.04
Arboreal omnivores	−0.25	0.39	0.27	0.35	−0.01	0.97
Nectarivores	−0.56	0.04	−0.52	0.06	0.20	0.49
**Observations**						
All species combined	−0.90	0.003	−0.93	0.001	0.93	0.001
Understory flocks	−0.72	0.05	−0.78	0.03	0.83	0.01
Canopy flocks	−0.81	0.02	−0.92	0.001	0.77	0.03
Army-ant followers	−0.64	0.08	−0.69	0.06	0.46	0.25
Gleaning insectivores	−0.83	0.01	−0.86	0.01	0.90	0.002
Sallying insectivores	−0.93	0.001	−0.91	0.002	0.93	0.001
Terrestrial insectivores	−0.71	0.05	−0.97	0.001	0.80	0.02
Bark insectivores	−0.80	0.02	−0.55	0.16	0.67	0.07
Dead leaf foragers	−0.52	0.18	−0.81	0.02	0.83	0.01
Arboreal omnivores	−0.85	0.007	−0.85	0.008	0.78	0.02
Arboreal frugivores	−0.82	0.012	−0.96	0.001	0.81	0.13
Terrestrial frugivores	−0.16	0.70	0.22	0.60	0.35	0.39
Arboreal granivores	−0.68	0.06	−0.82	0.013	0.75	0.03
Terrestrial granivores	−0.25	0.55	−0.54	0.16	0.64	0.09
Nectarivores	−0.68	0.06	−0.63	0.09	0.68	0.06

Overall observation rates also were correlated between plots (*r* = 0.93, *P* < 0.001) but showed no difference between plots (paired *t*-test, *t* = 0.9, df = 7, *P* = 0.40; Fig. 1B). Observation rates were correlated with capture rates on each plot (Harpia: *r* = 0.87, *P* < 0.005; Puma: *r* = 0.74, *P* < 0.05). Observation rates did not vary significantly from 2003 to 2006 (Fig. 1B) but were much lower in later years (correlations across all years combined Harpia: *r* = − 0.90, *P* < 0.005; Puma: *r* = − 0.93, *P* < 0.001); rates in 2014 were approximately half those observed during the first years of the study.

### Foraging guilds

**Figure 2:**
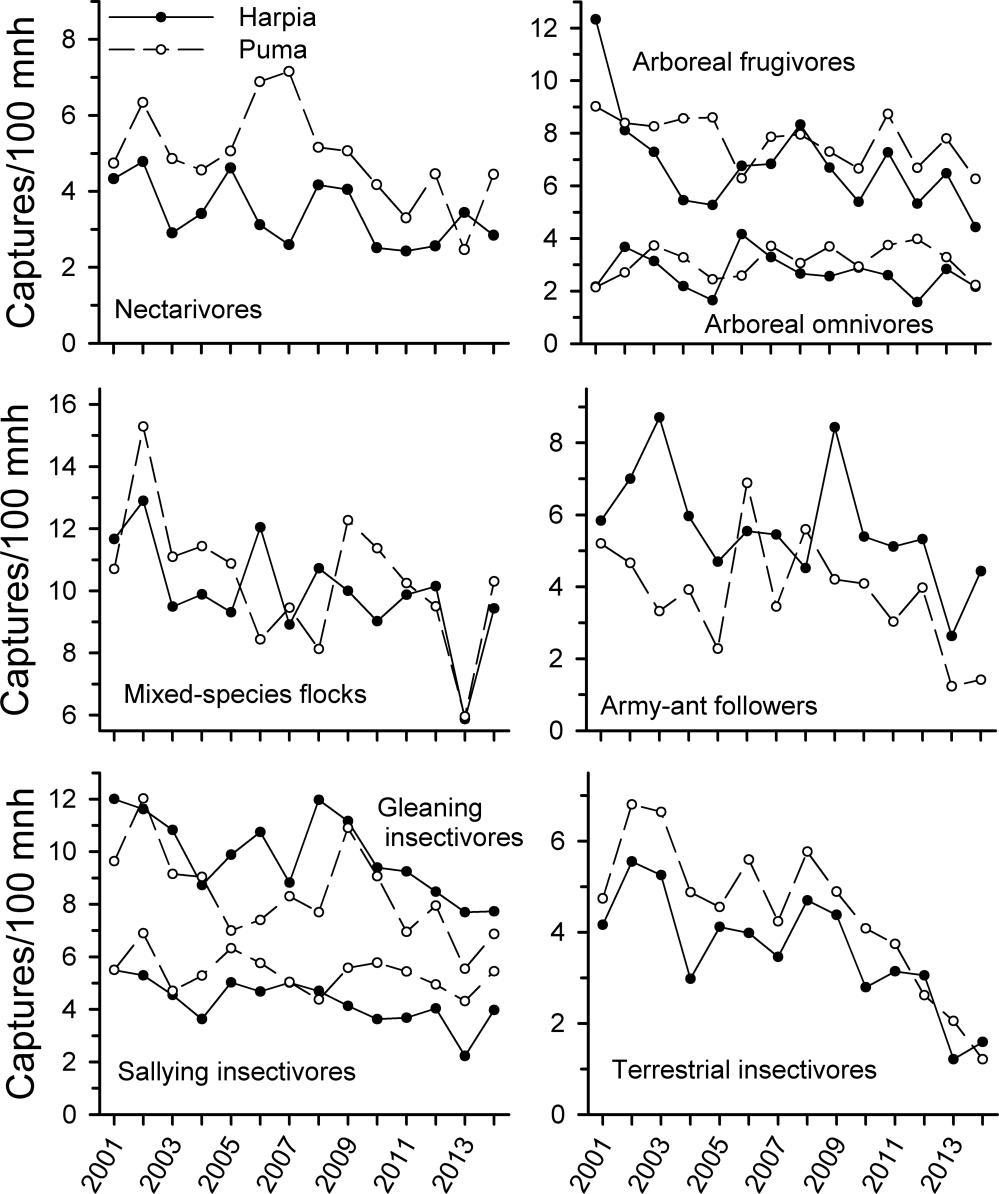
Capture rates of trophic guilds at Tiputini Biodiversity Station, Ecuador. Capture rates for different trophic guilds (from Terborgh et al., 1990) on two 100-ha plots (Harpia, Puma) at Tiputini Biodiversity Station, Ecuador, from 2001 to 2014.

Capture rates of all foraging guilds examined (except arboreal omnivores on Puma) declined from 2001 to 2014 (Fig. 2 and Table 1) but the extent of the decline varied among guilds and over time. Arboreal and terrestrial insectivores showed the steepest declines, particularly from 2009 to 2014; declines were not significant from 2001 to 2009 but were subsequently. Army-ant followers also showed steeper declines after 2009, with substantial fluctuations during the first years of the study. Mixed-species flocks also were quite variable but capture rates were substantially less in 2013 than in previous years; capture rates increased somewhat in 2014.

In contrast, frugivores declined in captures somewhat from 2001 to 2014 but unlike most insectivores, declines were not pronounced from 2009 on. Similarly, capture rates of nectarivores declined over time but correlations were much weaker than for insectivores. Correlations in capture rates between plots were strongest for terrestrial insectivores and somewhat less so for arboreal insectivores and mixed-species flocks; other guilds showed very different patterns between plots.

**Figure 3:**
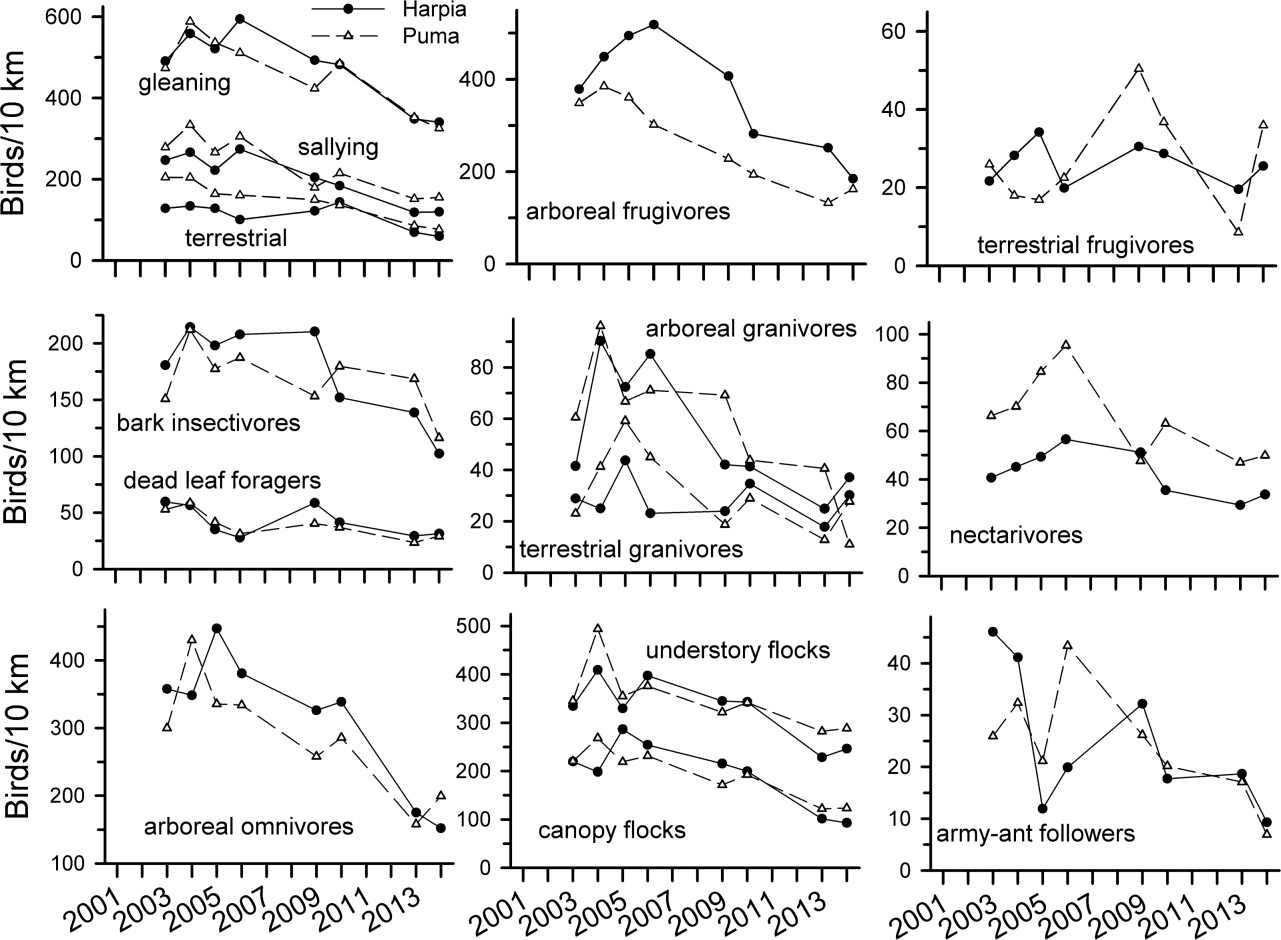
Observation rates for trophic guilds at Tiputini Biodiversity Station. Observation rates for different trophic guilds (from Terborgh et al., 1990) on two 100-ha plots (Harpia, Puma) at Tiputini Biodiversity Station, Ecuador, from 2001 to 2014.

Observations of most foraging guilds also declined over time (Fig. 3 and Table 1). Declines were particularly pronounced among most insectivores, arboreal omnivores, and arboreal granivores. Declines were not, however, as consistent among dead-leaf foragers, bark insectivores, terrestrial frugivores, and nectarivores. Both canopy and understory flocking species declined substantially whereas army-ant followers were more variable but nonetheless lower in later years. Patterns of observations were similar between the two plots for most guilds, with the exceptions of terrestrial frugivores, army-ant followers, and to a lesser extent, terrestrial granivores, bark insectivores, and nectarivores.

### Individual species

**Figure 4:**
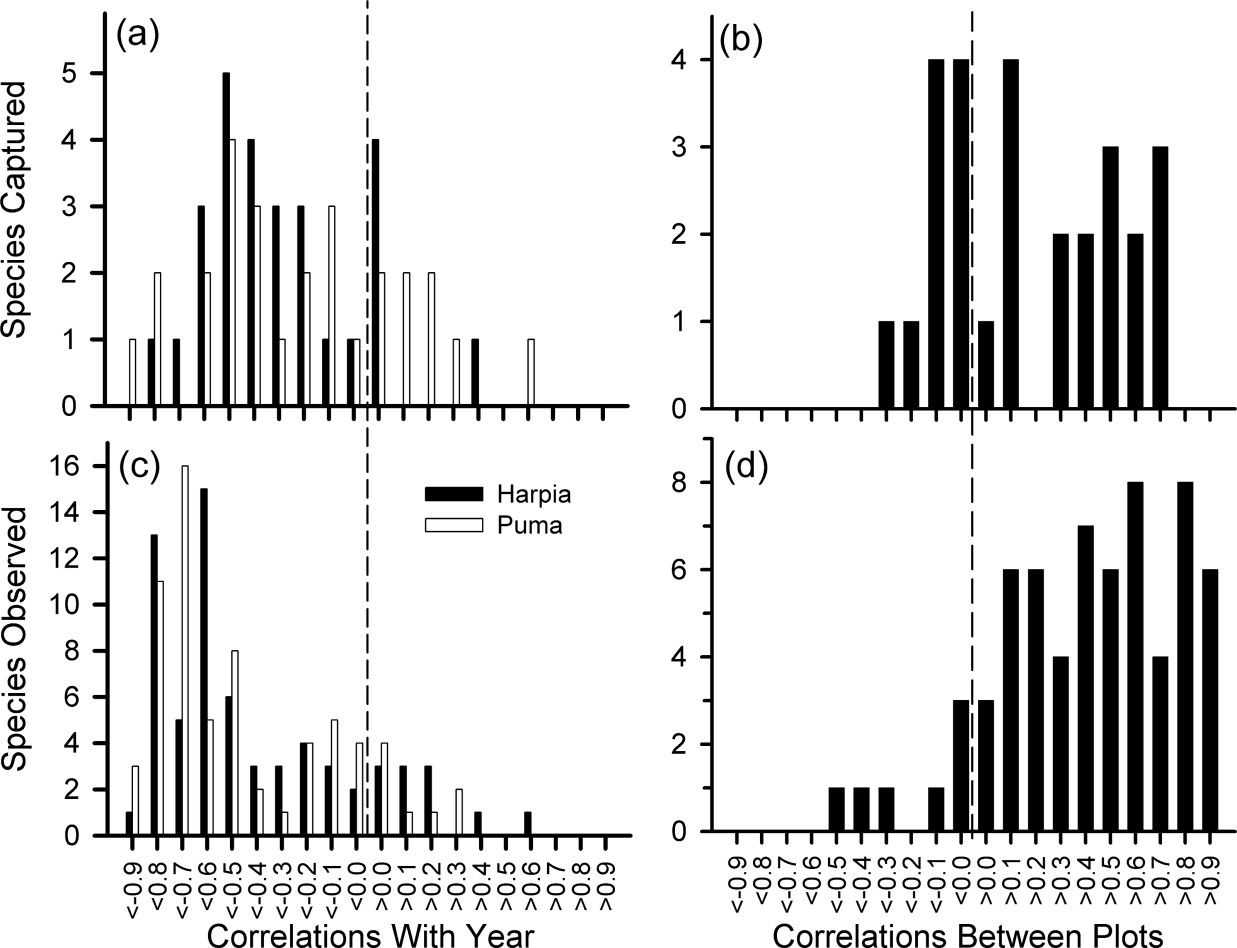
Frequency distribution of correlation coefficients. Number of species with different correlation coefficients (Pearson’s) between year and number of captures for 28 species (A) and numbers of observations for 68 species (C); distribution of correlation coefficients between plots based on captures (B) and observations (D).

We examined variation in 28 species represented by at least 140 captures. Although capture rates of most species declined over time (Fig. 4A), capture rates also varied from one year to the next, often substantially, and often showed different patterns between plots (Fig. 4B, see also Fig. S1 and Table S1). Declines in capture rates were particularly pronounced after about 2009 for many species (Fig. S1). Species that showed particularly pronounced declines on both plots included a frugivore (*Lepidothrix coronata*), terrestrial insectivores (*Sclerurus caudacutus*, *Formicarius* spp.), and understory insectivores (*Willisornis poecilinota*, *Myrmoborus myotherinus*); for these species, capture rates were correlated between plots (Table S1). In addition, several species showed large declines only on one plot: *Automolus infuscatus* (flocks) and *Hylophilus naevius* (understory insectivore) on Puma; *Glyphorynchus spirurus* (bark) and *Conopophaga peruviana* (ground) on Harpia. One understory flycatcher, *Platyrinchus coronatus*, declined on Harpia but increased on Puma. Thus, declines were, overall, most pronounced among insectivores.

We examined variation in 68 species or genera that were observed at least 140 times, or if less, at least 100 times on the plot examined (e.g., *Grallaria dignissima* was common on Puma but rarely seen on Harpia; the reverse was true for *Lipaugus vociferans*). Observations of most species declined over time and most were correlated between plots (Figs. 4C, 4D and Table S1). Species that showed significant declines included highly vocal and conspicuous species (e.g., *Ramphastos*), vocal but less conspicuous species (e.g., *Campylorhynchus turdinus*), and less vocal or conspicuous species (e.g., *Lepidothrix coronata*, *Euphonia* spp.) (Fig. S2).

### Southern oscillation index

**Figure 5:**
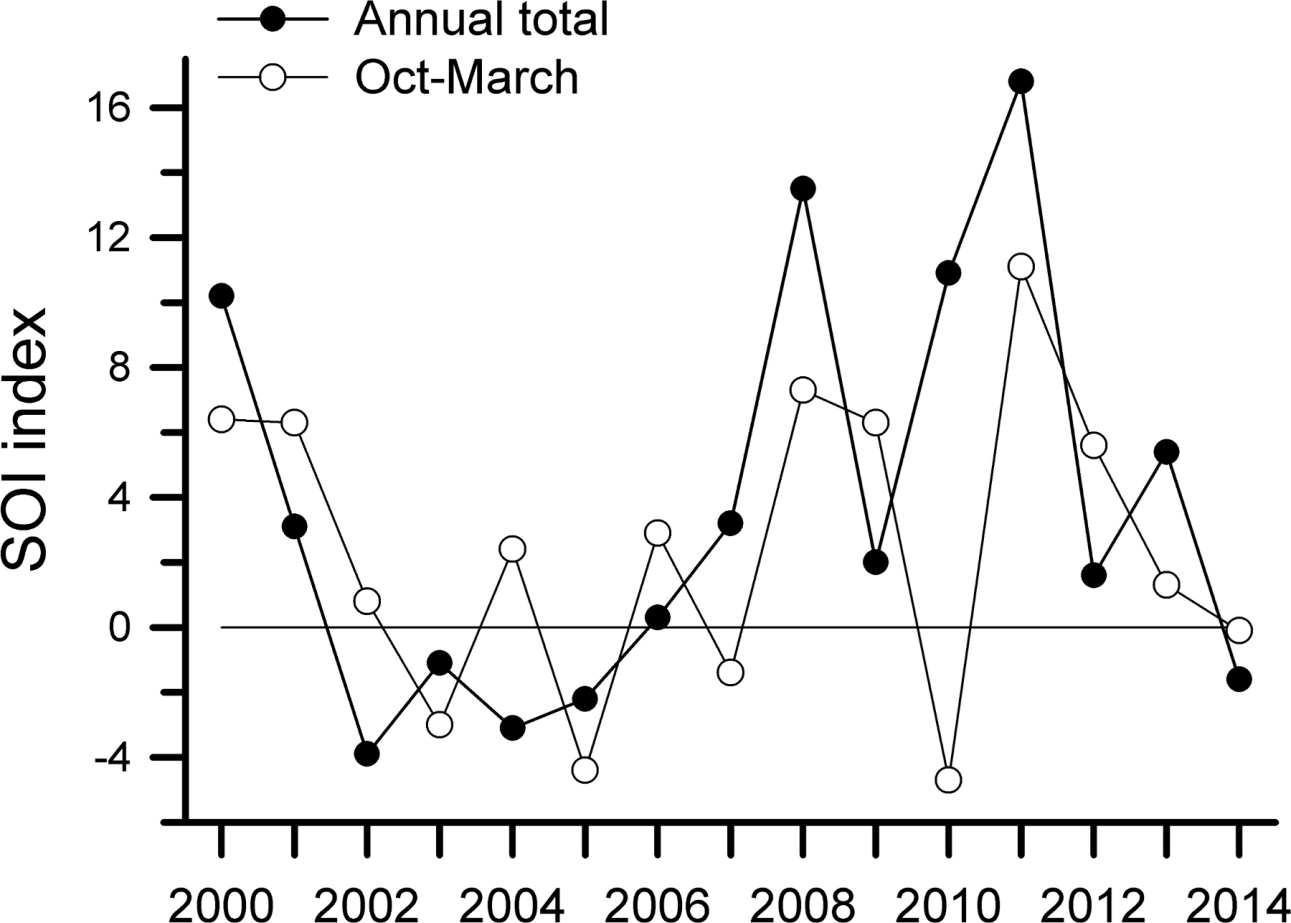
Southern Oscillation Index values. Southern Oscillation Index (SOI) values based on annual totals or on totals from Oct–Dec of previous year and Jan–Mar of current year. Positive values indicate La Niña-type events; negative values reflect El Niño.

Annual SOI values were lowest from 2002 to 2004 and highest from 2008 to 2011 (Fig. 5). Values for October through March, which encompasses the main breeding period of many species, followed a generally similar trend. Monthly values (Fig. S3) also demonstrated the tendency for SOI values to be greater in the later years of this study. We have both observation data and netting data for eight years; those rates were negatively correlated with SOI values for Oct–March, with a 2-yr time lag (*r* = − 0.62, −0.71, −0.71, and −0.60 for captures on Harpia, captures on Puma, observations on Harpia, and observations on Puma, respectively). Correlations with annual SOI values, with a 2-yr time lag, were weaker but still negative (−0.50, −0.50, −0.64, and −0.67, respectively). Capture rates were not correlated with SOI values when all 14 years were included.

## Discussion

Among the many hypotheses to explain the greater diversity of most organisms in the tropics (e.g., Pianka, 1966) is the idea that presumed greater climatic stability has allowed more species to coexist. Similarly, constant environmental conditions would be assumed to lead to constancy of resources and, hence, more stable populations, at least in areas unaffected by significant human activities. In contrast, in areas that have been substantially altered by humans (e.g., through deforestation, fragmentation, hunting), populations of many species have suffered significant declines in abundance (Robinson, 1999; Robinson, 2001; Sigel, Sherry & Young, 2006; Stouffer et al., 2011). A recent report assessing the state of vertebrate populations worldwide (World Wildlife Fund, 2014), concluded that abundance of vertebrates has declined by about 52% from 1970 to 2010; declines in the Neotropics were more pronounced than elsewhere. Primary causes for these declines were associated with habitat loss, with climate change implicated as the primary cause in 7.1% of species.

Few studies have examined population dynamics of birds in areas of the Neotropics where large expanses of lowland forests remain in relatively intact states (i.e., with little deforestation or fragmentation). Martinez & Gomez (2013) compared flock structure and abundance across an almost 20-year period in French Guiana, in an area largely unaffected by humans; flocks were remarkably stable across this period. Such constancy in abundance and spatial distribution was assumed to reflect relative constancy in resources. In contrast, species richness and capture rates of birds in a montane cloud forest reserve in Ecuador declined significantly over a 12-year period (1994/95 to 2006/07) (Latta et al., 2011). In the latter case, declines were most likely associated with habitat changes that occurred outside the relatively small (2,700 ha) reserve; climate change was a possible factor in declines of some species. Significant, long-term (>20 years) declines (by ∼two thirds) in abundance of winter residents (migrants) also were noted in a tract of high-quality tropical dry forest in Puerto Rico (Faaborg et al., 2013). Located within a biosphere reserve, the study area had not experienced recent habitat loss, suggesting that declines were a consequence of events during the breeding season. Survival rates during the winter period remained constant, suggesting that lack of recruitment may have been a factor contributing to the observed declines. Climatic factors (ENSO) also can influence breeding success and populations of Neotropical migrants, in both positive and negative ways (Sillett, Holmes & Sherry, 2000; Nott et al., 2002; Mazzerolle et al., 2005).

Protected areas often are established with the goal of protecting (or at least with the assumption of protection) species. Yet, as seen in montane forests (Latta et al., 2011) and tropical dry forest (Faaborg et al., 2013), factors extrinsic to reserves may negatively affect populations within such reserves. La Selva Biological Station, in Costa Rica, although connected to a relatively large national park (Braulio Carrillo National Park, 44,000 ha), has seen populations of many bird species decline, some to local extirpation, over the past few decades. Such declines likely are associated with changes in habitat and other conditions outside the park and station (Sigel, Sherry & Young, 2006). Global climate change has, in fact, been recognized as an important threat to the continued existence of both species and protected areas (Root et al., 2003; Hannah et al., 2007; World Wildlife Fund, 2014).

Unlike previous studies that have demonstrated population declines of birds in tropical forests affected by human activities, this study was conducted at a site located within a huge expanse of largely undisturbed lowland Amazonian forest, adjacent to Yasuní National Park and within Yasuní Biosphere Reserve. This study also is unique in that two relatively large plots (100 ha each) have been sampled for 14 years with two distinct but complementary methods: mist net captures and direct observations. The temporal and spatial scales provide an unparalleled perspective on dynamics of bird abundances in an area of relatively undisturbed lowland tropical forest located in the most biologically diverse region of the world (Bass et al., 2010). That bird populations in such an “untouched” area have apparently undergone substantial changes in numbers suggests that factors operating at large spatial scales can have important local effects. Yet, the idea that even large expanses of forest are not affected by human activities is almost certainly not true when considering large-scale climate patterns that may be affected by human activities (e.g., ENSO).

In contrast to the idea that bird populations in undisturbed lowland tropical forests should be relatively stable, at least in contrast to populations in areas that have experienced large-scale human impacts (Robinson, 2001; Stouffer et al., 2011), results of this study demonstrate that substantial changes in abundance across years is clearly possible. During the first years of this study, abundances of many species fluctuated but for most, fluctuations were not consistently up or down. Yet, in the years since 2008–2009, there has been a reasonably consistent decline in overall bird numbers as well as numbers of many individual species and trophic groups. Similarly, anecdotal reports from local guides and other visitors to the station provide support for the conclusion that bird numbers have declined in recent years.

Bird populations in a given site or region might decline as a consequence of habitat changes or loss both within a site or in nearby areas (Sigel, Sherry & Young, 2006; Latta et al., 2011), changes in availability of resources (e.g., Jones, Doran & Holmes, 2003), introduction or spread of disease (Warner, 1968; Van Riper III et al., 1986), introduction of new predators (Savidge, 1984) or changes in weather or climate (Stiles, 1992; Blake et al., 1994). Clearly, many of these possible factors could interact in various ways (e.g., climate change affecting plant phenology and production of flowers and fruits; weather changes causing fluctuations in insect numbers). Further, it is possible that sampling methods, including observer variation, might lead to the perception of changes in abundance even when numbers had not actually changed.

### Methodological considerations

Capture rates of birds often decline over time if an area is repeatedly netted, but those declines typically are seen over short time periods, such as when the same net locations are sampled for several days in a row (J Blake, pers. obs., 1985–1994). In our case, the same net locations were used each year but each net was opened for only one morning in January and one in March. Recapture rates are reasonably high (∼40%) suggesting that avoidance of nets is not a significant factor. Further, although capture rates declined somewhat during the first few years, those declines were offset by subsequent increases which would not be expected if netting *per se* had a long-term negative impact. Further, substantial declines (by about 50%) also were seen in observation data, which included data from many species that are not captured in nets.

All observations were done by one of us (JGB), so variation among observers is not an issue or possible cause of declines. If, on the other hand, ability to detect birds declined over time (i.e., because of loss of hearing) that might account for some of the observed declines. Yet, we would expect such changes to be most pronounced among species with high frequency songs that often are more difficult to detect as one gets older. In this study, increases in abundance were noted in some species with high frequency songs (e.g., *Platyrinchus coronatus*) and such species are still readily detected by JGB both in tropical and temperate forests (J Blake, pers. obs., 2015). Further, many of the species that have shown declines are loud and conspicuous, so a gradual decline in ability to detect such species seems an unlikely explanation. If uncontrolled variables related to environmental conditions or to observer abilities affect detection of species, we might expect that changes in abundance over time would not show a consistent pattern of decline but would, instead, vary in a more random fashion; such variation was not observed.

### Habitat effects

Declines are not likely to have occurred in response to habitat changes as there have been no substantial changes either at our study site or in surrounding regions during the course of this study. Tree falls and blowdowns are a normal and typical form of disturbance which might cause some small-scale shifts in spatial distribution of birds or other organisms. Yet, given that home ranges of most bird species in the area are larger than typical gaps, such disturbances would not be expected to cause large, consistent declines in bird populations at the scale of our study plots. Other forms of disturbance in eastern Ecuador are associated with oil exploration and extraction, which have increased in the general region of Yasuní (Finer et al., 2008). Such activities can have negative impacts on species as a consequence of road construction, noise, and other disturbances (Canaday, 1997). Yet, there has not been a substantial increase in such activity during the course of this study, at least in the area of our study plots. Similarly, roads may facilitate hunting by indigenous groups (e.g., Waorani) and hunting has caused declines in bird and mammal populations in some areas of Yasuní (Suárez et al., 2009). Although some hunting has occurred in the vicinity of the station (J Blake, pers. obs., 2014), the increase has not been substantial. Further, declines occurred in many species that are not targeted by hunters (e.g., *Lepidothrix, Formicarius*). Thus, we see no evidence that habitat loss or alteration, and associated human activities, may have caused the observed declines in bird numbers.

### Resource abundance

Responses of species to ENSO events may vary with foraging behavior and diet (Wolfe & Ralph, 2009). We do not, however, have concurrent measures of resource abundance (e.g., fruit, insects) so we cannot directly evaluate the potential importance of resource availability on populations. Yet, some results suggest that changes in resources may have played a role in at least some of the observed declines. Although declines in abundance occurred across a broad range of species and trophic guilds, declines in abundance were particularly pronounced among many insectivorous groups. Terrestrial insectivores, for example, showed particularly pronounced declines in abundance; such insectivores also are among those most negatively affected by habitat fragmentation (Powell, Cordeiro & Stratford, 2015). Disappearance from small patches of habitat may be related to reductions in availability of leaf-litter insects, suggesting that such species are sensitive to loss of resources. Understory insectivores often are particularly vulnerable to disturbances (Powell, Cordeiro & Stratford, 2015) and many also declined in abundance in this study. Changes in precipitation can affect understory insectivores in various direct and indirect ways through effects on resource (insect) abundance (Powell, Cordeiro & Stratford, 2015), which might account for some of the observed declines. Declines were less general among most frugivores and nectarivores, suggesting that resources fluctuate in different ways. Lack of sufficient resources might lead to reductions in either nesting attempts or nesting success, with consequences for recruitment. When combined with typical survival rates of 3 to 5 years for many species in the understory (Blake & Loiselle, 2013), lack of recruitment could lead to subsequent declines in overall abundance.

### Disease

Blood parasites such as *Plasmodium* and *Haemoproteus* (haemosporidia) and other diseases (e.g., West Nile virus) can be harmful to their hosts (Van Riper III et al., 1986; Merino et al., 2000) and, in some cases, can cause large-scale population declines and even extinction (e.g., *Plasmodium relictum* and Hawaiian native avifauna; Warner (1968); Van Riper III et al. (1986). In a previous study based on data collected from our study plots (Svensson-Coelho et al., 2013), overall prevalence of blood parasites among species ranged from a low of 5.6% in *Pipra filicauda* to 91.2% in *Formicarius colma*. All well-sampled species were infected, suggesting that infection is widespread among species at our site. Further, prevalence varied among years, from an overall prevalence of 14.5% in 2006 to a high of 33% in 2009; the increase in 2009 was widespread across species but was followed by a decline in overall prevalence in 2010. If the increase in infection rates had negative consequences for reproduction or survival, this might account for some of the declines. Although low level, chronic infections are generally considered benign, chronic malaria can, in some cases, have negative consequences for lifespan and reproductive success (Asghar et al., 2015). Several species with particularly high infection rates, such as *Automolus infuscatus* (59%), *Formicarius analis* (54%), and *Formicarius colma* (91%) (Svensson-Coelho et al., 2013), were among those species that showed some of the steepest declines in abundance. *Lepidothrix coronata* (16.3%) had the highest prevalence among manakins and also showed the greatest decline in abundance among members of that family. Yet, some species with relatively low infection rates, such as *Pithys alba* (6%) and *Willisornis poecilinota* (24%), also showed substantial declines in abundance. Increased levels of infection might make some individuals more susceptible to other factors, such as reduced availability of resources, leading to population declines. At present, we do not have any direct evidence, however, to link disease to declining numbers of any particular species.

### Climate

Warming temperatures cause species to move to higher elevations (Forero-Medina et al., 2011; Anderson et al., 2013; Freeman & Freeman, 2014), which could lead to declines in abundance at lower elevations. If temperature increases in lowland forest are sufficient to affect reproduction and survival of many species, then populations could certainly decline, particularly given that there would be little opportunity to move to higher elevations or latitudes (see Colwell et al., 2008). Global temperature has apparently increased at a rate of ∼0.2 °C/decade since 1975 (Hansen et al., 2006) with an increase of ∼0.25°C/decade in lowland tropics (cited in Colwell et al., 2008). If correct, then we might expect to see increases of ∼0.12 to ∼0.15°C during 5–7 years (i.e., from 2008 to 2014). It is unlikely that such an increase would cause the rapid population declines seen in this study. Thus, we do not think that warming temperatures have been the cause of the observed changes.

What has changed during the course of this study has been the strength and frequency of La Niña events, as shown by variation in SOI. Particularly pronounced events occurred in the latter part of the study and show some correlation with population declines. Extreme La Niña events, such as that of 2010–2011, can bring excessive rains, which can cause widespread damage from flooding (e.g., Hoyos et al., 2013; see also Restrepo & Kjerfve, 2000) and which might also affect reproductive behavior of birds. Changes in rainfall associated with changes in climate might affect birds directly (through effects on reproductive success; Stiles, 1992) or indirectly through effects on resource abundance, body condition, predation, or susceptibility to disease. Reduction in reproductive success would lead to population declines as adult birds die and are not replaced with new recruits. In lowland tropical wet forests, such as our site, nest failure is already high (Ryder et al., 2008), so further reductions in nesting attempts or success might be expected to have noticeable effects on recruitment. Previous studies have noted changes in reproductive success, survival, and abundance associated with changes in global circulation patterns (e.g., LaManna et al., 2012; García-Pérez et al., 2014). In our area, stronger La Niña events are associated with increased rainfall during the period of time when many birds typically are nesting, and when our samples (nets and observations) occur. Given that observed declines in abundance of many species coincided with a period with considerably higher SOI values (La Niña events), it is at least plausible that this change in climate patterns could account for observed declines in bird populations. Although the mechanism through which climate change acts is yet unknown, if changes in climate are indeed driving population declines, then as conditions change in the future (if they do), we might expect to see an increase in bird populations over time.

## Supplemental Information

10.7717/peerj.1177/supp-1 Table S1 Correlations in capture rates and observation rates of individual species at Tiputini Biodiversity Station, Ecuador Correlations in capture rates and observation rates of individual species at Tiputini Biodiversity Station, Ecuador, across years (2001–2014) and between plots (Harpia, Puma). *P*-values are given without any correction for multiple comparisons.

10.7717/peerj.1177/supp-2 Figure S1 Capture rates for selected bird species atTiputini Biodiversity Station, Ecuador, from 2001 to 2014 Capture rates (number of birds captured/100 mist-net-hours) for selected species on two 100-ha plots (Harpia, Puma) at Tiputini Biodiversity Station, Ecuador, from 2001 to 2014.

10.7717/peerj.1177/supp-3 Figure S2 Observation rates for selected bird species Tiputini Biodiversity Station, Ecuador, from 2001 to 2014 Observation rates (numbers of birds observed per 10 km of transect) for selected species on two 100-ha plots (Harpia, Puma) at Tiputini Biodiversity Station, Ecuador, from 2001 to 2014.

10.7717/peerj.1177/supp-4 Figure S3 Monthly Southern Oscillation Index (SOI) values. Positive values indicate La Niña-type events; negative values reflect El Niño.

10.7717/peerj.1177/supp-5 Supplemental Information 5 Numbers of mist net captures, by plot and year List of species captured on two plots at Tiputini Biodiversity Station, Ecuador, 2001–2014. Total numbers of captures are given by plot and year for each species.

10.7717/peerj.1177/supp-6 Supplemental Information 6 Numbers of observations per species, by plot and year List of species observed on two plots at Tiputini Biodiversity Station, Ecuador, 2003–2014. Total numbers of observations are given by plot and year for each species.

## Additional Information and Declarations

### Field Study Permissions

The following information was supplied relating to field study approvals (i.e., approving body and any reference numbers): Work at Tiputini Biodiversity Station was conducted in accordance with research permit number 13-IC-FAU-DFN (and subsequent renewals), Ministerio del Ambiente, Distrito Forestal Napo, Tena, Ecuador.
