# Supplementary material for: Enigmatic declines in bird numbers in lowland forest of eastern Ecuador may be a consequence of climate change
Source: PeerJ. 2015 Aug 11;3:e1177. doi: 10.7717/peerj.1177 (PMC4558082; doi:10.7717/peerj.1177)
Supplement: Table S1 — Correlations in capture rates and observation rates of individual species at Tiputini Biodiversity Station, Ecuador, across years (2001–2014) and between plots (Harpia, Puma). P-values are given without any correction for multiple comparisons. [file peerj-03-1177-s001.docx]

Table S1. Correlations in capture rates and observation rates of individual species at Tiputini Biodiversity Station, Ecuador, across years (2001-2014) and between plots (Harpia, Puma). *P*-values are given without any correction for multiple comparisons.

___________________________________________________________________________________

Across years Between plots

_________________________ ______________

Group Harpia Puma

____________ __________

r P< r P< r P<

___________________________________________________________________________________

Captures

*Geotrygon montana* 0.23 0.44 -0.01 0.98 0.39 0.16

*Phaethornis boucieri* -0.20 0.50 -0.54 0.05 0.30 0.30

*Phaethornis malaris* -0.65 0.02 -0.52 0.06 0.56 0.04

*Automolus infuscatus* -0.09 0.76 -0.65 0.02 0.45 0.11

*Philydor erythrura* -0.31 0.28 -0.45 0.10 -0.11 0.72

*Sclerurus caudacutus* -0.70 0.005 -0.81 0.001 0.65 0.02

*Glyphorynchus spirurus* -0.69 0.007 -0.11 0.70 0.54 0.05

*Xiphorhynchus ocellatus* -0.41 0.15 -0.17 0.56 -0.16 0.59

*Pithys alba* 0.03 0.91 -0.34 0.24 -0.01 0.98

*Gymnopithys leucaspis* -0.51 0.06 -0.51 0.06 0.51 0.06

*Hylophylax naevia* -0.42 0.14 -0.64 0.02 0.18 0.55

*Willisornis poecilinota* -0.59 0.03 -0.46 0.10 0.79 0.001

*Thamnomanes ardesiacus* 0.00 0.99 -0.47 0.09 0.06 0.84

*Thamnomanes caesius* -0.30 0.30 0.36 0.21 -0.26 0.37

*Myrmoborus myotherinus* -0.59 0.03 -0.94 0.001 0.64 0.02

*Epinecrophylla fjeldsaii* 0.47 0.09 0.19 0.51 -0.05 0.88

*Myrmotherula axillaris* -0.41 0.15 0.12 0.67 0.30 0.29

*Myrmotherula hauxwelli* 0.05 0.87 0.09 0.76 0.12 0.69

*Myrmotherula longipennis* -0.34 0.23 0.02 0.95 -0.05 0.86

*Formicarius* spp. -0.59 0.03 -0.80 0.001 0.71 0.005

*Conopophaga peruviana* -0.83 0.001 -0.10 0.72 0.15 0.61

*Mionectes oleagineus* -0.46 0.10 0.25 0.38 0.10 0.74

*Platyrinchus coronatus* -0.60 0.02 0.67 0.009 -0.13 0.65

*Chiroxiphia pareola* -0.22 0.46 -0.28 0.33 0.42 0.13

*Lepidothrix coronata* -0.72 0.004 -0.60 0.03 0.73 0.004

*Pipra filicauda* 0.04 0.90 -0.17 0.57 -0.12 0.69

*Dixiphia pipra* -0.29 0.31 -0.26 0.36 -0.04 0.89

*Turdus albicollis* -0.36 0.21 0.27 0.34 -0.36 0.20

Observations

*Tinamus* spp. 0.14 0.74 -0.14 0.73 0.81 0.02

*Crypturellus* spp. -0.52 0.19 -0.73 0.05 0.74 0.05

*Patagioenas* spp. -0.62 0.10 -0.87 0.005 0.33 0.42

*Geotrygon montana* 0.29 0.48 0.03 0.94 0.34 0.41

*Amazona* spp. -0.21 0.61 -0.75 0.05 0.65 0.08

*Phaethornis malaris* -0.29 0.49 -0.56 0.15 0.48 0.23

*Trogon* spp. -0.82 0.02 -0.77 0.03 0.89 0.003

*Baryphthengus martii* -0.87 0.005 -0.86 0.007 0.83 0.02

*Monasa morphoeus* -0.66 0.08 -0.67 0.07 0.54 0.17

*Capito niger* -0.63 0.10 -0.85 0.004 0.80 0.02

*Ramphastos tucanus* -0.77 0.03 -0.81 0.02 0.90 0.003

*Ramphastos vitellinus* -0.35 0.40 -0.81 0.02 0.60 0.12

*Pteroglossus* spp. -0.56 0.15 -0.76 0.03 0.26 0.53

*Selenidera reinwartii* -0.68 0.07 -0.89 0.003 0.65 0.08

*Celeus grammicus* -0.84 0.01 -0.55 0.16 0.50 0.21

*Melanerpes cruentatus* -0.81 0.02 -0.79 0.02 0.65 0.08

*Campephilus* spp. -0.17 0.68 0.37 0.36 -0.02 0.96

*Piculus* spp. -0.59 0.12 -0.11 0.80 0.75 0.03

*Automolus infuscatus* -0.67 0.07 -0.61 0.10 0.93 0.001

*Automolus subulatus* 0.66 0.08 -0.47 0.24 -0.12 0.78

*Ancistrops strigilatus* -0.85 0.009 -0.93 0.001 0.87 0.005

*Xiphorhychus guttatus* -0.88 0.005 -0.79 0.021 0.93 0.007

*Glyphorynchus spirurus* -0.36 0.38 -0.02 0.95 -0.04 0.92

*Cercomacra cinerescens* -0.80 0.02 -0.71 0.06 0.50 0.21

*Cymbilaimus lineatus* 0.29 0.48 -0.02 0.97 -0.59 0.12

*Formicarius* spp. -0.78 0.03 -0.85 0.008 0.77 0.03

*Chamaeza nobilis* -0.70 0.06 -0.74 0.04 0.43 0.29

*Grallaria dignissima* -0.58 0.13

*Myrmothera campanisoma* 0.16 0.71 -0.82 0.02 0.14 0.74

*Hypocnemis cantator* -0.34 0.41 -0.76 0.03 0.61 0.11

*Willisornis poecilinota* -0.49 0.22 0.07 0.87 0.46 0.25

*Hylophylax naevia* 0.00 0.99 -0.26 0.54 0.10 0.81

*Myrmeciza fortis* 0.16 0.70 -0.76 0.03 0.26 0.54

*Epinecrophylla erythrura* 0.09 0.84 -0.37 0.37 0.07 0.87

*Myrmetherula axillaris* -0.84 0.009 -0.16 0.70 0.24 0.57

*Myrmetherula brachyura* -0.08 0.85 -0.29 0.49 0.92 0.002

*Myrmetherula longipennis* 0.40 0.32 0.04 0.93 0.08 0.86

*Myrmetherula menetriesii* -0.29 0.49 -0.56 0.15 0.66 0.08

*Myrmoborus myotherinus* 0.06 0.89 -0.72 0.05 -0.48 0.23

*Thamnomanes caesius* -0.63 0.10 -0.53 0.17 0.59 0.12

*Thamnomanes ardesiacus* -0.80 0.02 -0.78 0.03 0.82 0.02

*Thamnophilus* spp. -0.68 0.06 -0.51 0.19 0.60 0.11

*Pygiptila stellaris* -0.21 0.62 -0.24 0.56 0.26 0.53

*Liosceles thoracicus* -0.76 0.03 -0.59 0.13 0.47 0.24

*Mionectes oleagineus* -0.06 0.88 -0.68 0.07 -0.02 0.96

*Lophotriccus vitiosus* -0.67 0.07 -0.84 0.01 0.69 0.06

*Lipaugus vociferans* -0.67 0.07

*Querula purpurata* -0.68 0.06 -0.41 0.32 0.21 0.62

*Lepidothrix coronata* -0.91 0.001 -0.95 0.001 0.93 0.001

*Pipra pipra* -0.74 0.04 0.15 0.73 0.25 0.56

*Pipra filicauda* -0.89 0.009

*Ceratopipra erythrocephala* -0.44 0.27 -0.75 0.05 0.18 0.66

*Chiroxiphia pareola* -0.19 0.66 -0.79 0.02 0.36 0.38

*Machaeropterus regulus* -0.69 0.06 0.30 0.47 0.17 0.69

*Tyranneutes stolzmani* -0.86 0.007 -0.76 0.03 0.61 0.11

*Hylophilus hypoxantha* -0.64 0.09 -0.13 0.76 0.48 0.23

*Turdus lawrencii* -0.81 0.02 -0.70 0.06 0.62 0.11

*Turdus albicollis* -0.57 0.14 0.35 0.40 -0.38 0.35

*Ramphocaenus melanurus* -0.10 0.80 0.05 0.90 0.19 0.66

*Henecorhina leucosticta* -0.65 0.08 -0.68 0.06 0.46 0.25

*Campylorhynchus turdinus* -0.89 0.003 -0.95 0.001 0.91 0.002

*Microcerculus marginatus* -0.56 0.15 -0.03 0.94 0.19 0.65

*Pheugopedius coraya* -0.50 0.20 -0.18 0.68 0.49 0.22

*Dacnis* spp. -0.70 0.05 -0.54 0.16 0.75 0.05

*Euphonia* spp. -0.82 0.02 -0.85 0.001 0.84 0.01

*Saltator grossus* -0.75 0.04 -0.04 0.91 0.03 0.95

*Psaracolius* spp. -0.53 0.18 -0.71 0.06 0.87 0.005

*Cacicus cela* 0.24 0.57 -0.23 0.58 0.32 0.44
