# Supplementary material for: Enigmatic declines in bird numbers in lowland forest of eastern Ecuador may be a consequence of climate change
Source: PeerJ. 2015 Aug 11;3:e1177. doi: 10.7717/peerj.1177 (PMC4558082; doi:10.7717/peerj.1177)
Supplement: Figure S1 — Capture rates (number of birds captured/100 mist-net-hours) for selected species on two 100-ha plots (Harpia, Puma) at Tiputini Biodiversity Station, Ecuador, from 2001 to 2014. [file peerj-03-1177-s002.pdf]

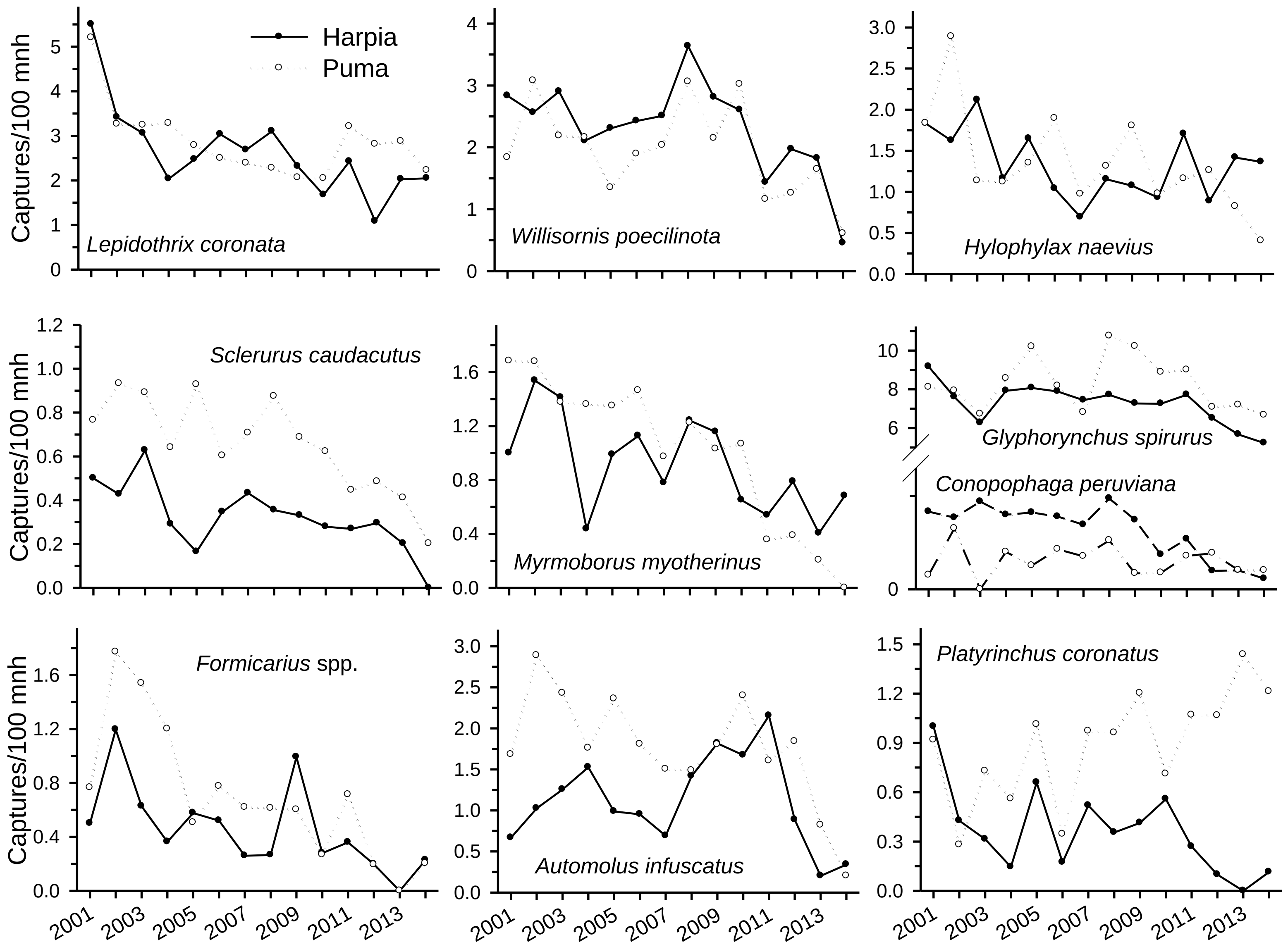

Figure S1. Capture rates (number of birds captured/100 mist-net-hours) for selected species on two 100-ha plots (Harpia, Puma) at Tiputini Biodiversity Station, Ecuador, from 2001 to 2014.
