# Supplementary material for: Enigmatic declines in bird numbers in lowland forest of eastern Ecuador may be a consequence of climate change
Source: PeerJ. 2015 Aug 11;3:e1177. doi: 10.7717/peerj.1177 (PMC4558082; doi:10.7717/peerj.1177)
Supplement: Figure S3 — Positive values indicate La Niña-type events; negative values reflect El Niño. [file peerj-03-1177-s004.pdf]

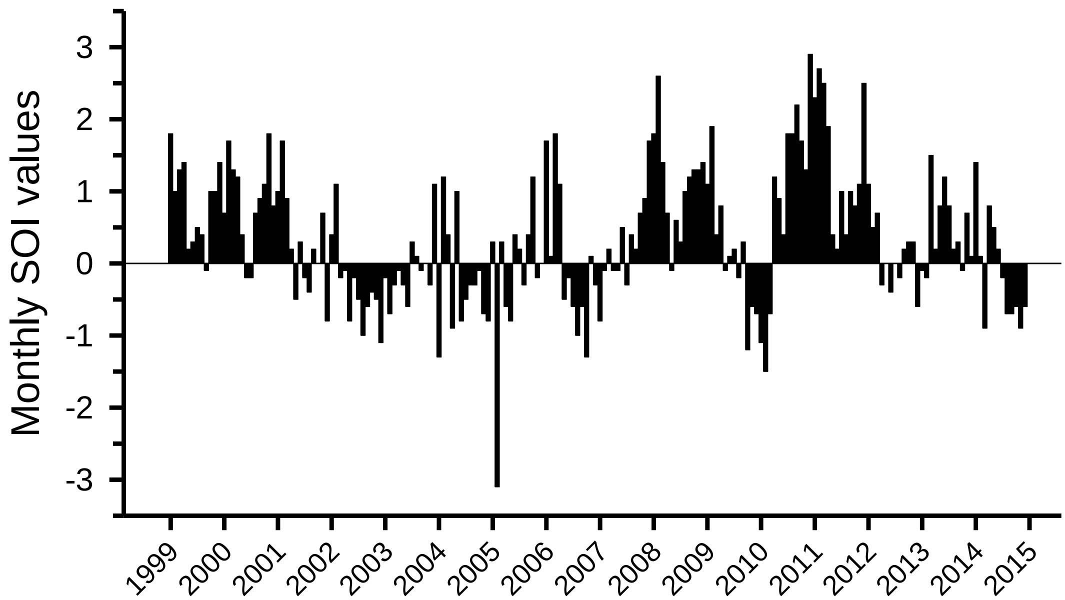

Figure S3. Monthly Southern Oscillation Index (SOI) values. Positive values indicate La Niña-type events; negative values reflect El Niño.
